# Comparison of 'Excision and Primary Repair' with 'Bascom's Technique' in the Surgical Treatment of Pilonidal Sinus

**DOI:** 10.7759/cureus.7338

**Published:** 2020-03-20

**Authors:** Muhammad Osman Karim, Kashuf A Khan, Abdul Jalil Khan, Syed Hussain Abbas, Omer Abdalla, Muhammad Imran Aslam

**Affiliations:** 1 General Surgery, Royal Shrewsbury and Telford Hospital National Health Service (NHS) Trust, Shrewsbury, GBR; 2 Family Medicine, Khyber Medical University, Peshawar, PAK; 3 General Practice, National Health Service (NHS), Bolton, GBR; 4 General Surgery, Buckinghamshire Healthcare NHS Trust, Wycombe Hospital, High Wycombe, GBR; 5 General Surgery, Warwick Hospital, Warwick, GBR

**Keywords:** pilonidal sinus, excision & primary repair, bascom's repair

## Abstract

Introduction

The management of the chronic pilonidal disease is variable and the principles of treatment are aimed to eradicate the sinus tract, promote wound healing, prevent disease recurrence, and improve the quality of life of the patient. This study aims to compare the effectiveness of excision and primary closure, and Bascom’s technique in the management of pilonidal sinus disease.

Methods

The study was performed at a tertiary hospital from April to October 2011. All patients with chronic pilonidal sinus were included in the study (n=60) and randomly allocated into Group A - undergoing excision and primary closure (n=30) and Group B - undergoing Bascom’s repair. Comparative outcomes of interest were duration of hospital admission, post-operative pain, wound infection, wound-healing and disease recurrence.

Results

The mean age of presentation was 24.18±5.6 years. A higher number of patients in Group A were discharged within 24 hours compared to Group B (p = 0.001). Group B had significantly less post-operative pain by the first postoperative week (p = 0.049). Group B had lower infection rates with clean wounds observed in 28 (93.3%) patients compared to 23 (76.7%) in Group A by the first postoperative week (p = 0.07). Recurrence rate during 12-week follow-up was observed in one (3.3%) patient in Group B, and five (16.7%) in Group A (p= 0.085).

Conclusions

Patients who underwent Bascom's operation had less postoperative pain, lower infection rates and disease recurrence, and better wound healing. Therefore, in our patient cohort, we conclude Bascom’s repair appears to be superior to primary excision and repair in reducing patient morbidity.

## Introduction

Pilonidal sinus disease is characterized by an epithelial tract situated in the skin of the natal cleft, a short distance behind the anus and generally contains hair follicles [1]. It poses a considerable amount of morbidity and additional healthcare costs in young adults due to discomfort, time off work and particularly when complications such as abscess formation or a draining sinus tract ensue [2, 3]. The incidence is 10 times more common in males due to their hirsute nature and affects adults in their second or third decade of life. Those of Caucasian origin are more likely to suffer from Pilonidal sinus disease than those of African origin due to different hair characteristics and growth patterns [4]. Pilonidal sinus disease is extremely uncommon both before puberty and as well as after the age of 40 years. [5]

No current treatment method satisfies all the requirements for the management of the chronic pilonidal disease. However, the principles of treatment are aimed to eradicate the sinus tract, promote wound healing of overlying skin and minimize disease recurrence as well as patient morbidity [6]. Management techniques used in treating pilonidal sinus disease include phenol injection with or without local excision of the sinus, incision and drainage of the pilonidal abscess, excision and healing by secondary intention, excision and primary closure, excision and reconstructive flaps such as Karydakis procedure and Bascom’s technique [7, 8,9,10, 11].

Overall, the two commonly used techniques for the treatment of pilonidal sinus disease include excision and primary closure and Bascom’s operation. In excision and primary closure, all the sinus tracts (midline as well as lateral) are excised by an elliptical incision around the sinuses followed by the closure of the wound primarily. This can be done with or without a drain. The closure of the wound is more cosmetically acceptable for some patients and is associated with shorter healing time and this reduced time off work [10]. However, Bascom’s operation involves incision, drainage and curettage of a chronic abscess through a lateral incision. Additionally, the excision of midline pits with only a minimal amount of surrounding tissue is performed [12]. The lateral wound is then left open and the midline incisions are closed [8]. There are several advantages of Bascom’s technique compared to other procedures including low recurrence rate, minimal morbidity, suitability for day surgery and considerable financial savings [12]. We, therefore, aimed to compare the effectiveness of excision and primary closure, and Bascom’s technique in the treatment of pilonidal sinus disease. The main outcomes assessed included duration of hospital admission, postoperative pain, wound infection, wound-healing and disease recurrence.

## Materials and methods

The study is based on an interventional quasi-experimental design with non-probability prospective sampling and was conducted from April 2011 to October 2011 at The Jinnah Teaching Hospital, Lahore, Pakistan. The study was registered with the institution and with the Research Registry under, "researchregistry2154" and received the necessary institutional approval.

All patients with a clinical diagnosis of chronic pilonidal sinus disease were included in the study. The criterion for exclusion included the site of the pilonidal sinus disease other than midline coccyx, patients with acute exacerbation of sinus disease (abscess formation) and concurrent medical problems, patients unfit for anesthesia, uncontrolled diabetes mellitus and recurrent pilonidal sinus disease. A total of 60 patients who met the inclusion criteria were selected from the outpatient department and included in the study. They were randomly allocated equally into two groups with the assistance of SPSS random number generator. Group A consisted of 30 patients who underwent excision and primary closure, and Group B consisted of 30 patients who underwent Bascom’s repair. All patients included in the study underwent pre-operative assessment with baseline investigation and were subsequently consented and booked for an elective operation with prophylactic antibiotics given on the day. The operative procedures were performed by the consultants. Clear post-operative instructions were given to every patient regarding daily dressing of the wound, sitz bath and time for stitches removal. All patients were followed-up at one week, three weeks and 12 weeks post-operatively with outcome data collected on a standardized proforma.

The collected data was analyzed using SPSS software. The primary outcomes including duration of admission, post-operative pain, wound healing and recurrence were presented as mean, standard deviations, frequency percentages. The differences between the two groups were compared using the chi-square test and p-value <0.05 was considered statistically significant.

## Results

Out of the 60 patients with a diagnosis of pilonidal sinus included in study 55 (91.7%) were male and five (8.3%) were female. The mean age of presentation was 24.18±5.6, the median was 23.50 and the range was 14-40 years. The majority of patients 43 (71.7%) were aged between 20- 30 years at the time of diagnosis (Table 1). The occupation of all the patients was taken into account. The majority of 47 (78.4%) patients had prolonged sitting and sedentary jobs. Nineteen (31.7%) were students, 12 (20%) were drivers and 16 (26.7%) were shopkeepers. However, only 13 (21.6%) patients had other non-sedentary professions.

**Table 1:** Patient demographics

		n (%)
Age (years)	≤20	11 (18.3)
	20-30	43 (71.7)
	≥30	6 (10)
Sex	Male	55 (91.7)
	Female	5 (8.3)

All the patients were discharged providing that there were no immediate complications, wound review was satisfactory and pain responded to simple analgesia. In Group A, 21 (70%) patients were discharged within 24 hours, and nine (30%) patients were discharged within 48 hours. In Group B, 11 (36.7%) were discharged within 24 hours and 19 (63.3%) patients were discharged within 48 hours. The chi-square value was 6.696 and p-value = 0.010. All the patients were followed-up at one-week post-operatively to assess pain. In Group A, 15 (50%) patients reported no pain, 12 (40%) reported moderate pain and only three (10%) expressed severe pain. In Group B, 23 (76.7%) patients reported no pain; and moderate pain was experienced in only seven (23.3%) cases. No patient in Group B reported severe pain at one week post-operatively. The chi-square value was 6 and p-value = 0.049. Surgical wounds were also examined at one-week post-operatively for signs of infection. In Group A, 23 (76.7%) patients had clean wounds and seven (23.3%) patients were found with infected wounds. In Group B, 28 (93.3%) patients had clean wounds and two (6.7%) patients had signs of wound infection. The chi-square value was 3.268 and p-value = 0.070. At the three-week follow-up wounds were reviewed to assess for healing. In Group A, 24 (80%) patients had healed wounds and 6 (20%) patients had partially healed wounds. In Group B, five (16.7%) patients had completely healed wounds and 24 (80%) patients showed signs of partial wound healing. There was only one (3.3%) patient whose wound had not healed completely at three weeks postoperatively. Chi-Square value was 24.248 and p-value = 0.00001. Additionally, wounds were further assessed for signs of infection. In Group A, 25 (83.3%) patients had with clean wounds and five (16.7%) patients were found with infected wounds. In Group B, 29 (96.7%) patients had clean wounds and only one (3.3%) patient had signs of infection. The chi-square value was 2.963 and p-value = 0.085. All the patients were followed-up after 12 weeks post-operatively to assess for disease recurrence. In Group A, five (16.7%) patients were found to have the recurrent disease compared to only one (3.3%) patient in Group B. The chi-square value was 2.963 and p-value = 0.085 (Table 2).

**Table 2:** Primary Outcomes

Primary Outcomes		Outcome Variables n (%)				P Value
Duration of Admission		0-24 hours		24-48 hours		
	Group A	21/30 (70)		9/30 (30)		0.001
	Group B	11/30 (36.7)		19/30 (63.3)		
Pain (one week)		No Pain	Moderate		Severe	
	Group A	15/30 (50)	12/30 (40)		3/30 (30)	0.049
	Group B	3/30 (30)	3/30 (30)		3/30 (30)	
Wound (one week)		Clean		Infected		
	Group A	23/30 (76.7)		7/30 (23.3)		0.07
	Group B	28/30 (93.3)		2/30 (93.3)		
Healing (three weeks)		Not healed	Partially		Healed	
	Group A	0/30 (0)	6/30 (20)		24/30 (80)	0.00001
	Group B	1/30 (3.3)	24/30 (80)		5/30 (16.7)	
Wound (three weeks)		Clean		Infected		
	Group A	25/30 (83.3)		5/30 (16.7)		0.085
	Group B	39/30 (96.7)		1/30 (3.3)		
Recurrence (12 weeks)		Present		Absent		
	Group A	5/30 (1.7)		25/30 (83.3)		0.085
	Group B	1/30 (3.3)		29/30 (96.7)		

## Discussion

Pilonidal sinus disease is an acquired condition usually seen in young adults and is associated with high postoperative morbidity. The onset of pilonidal sinus disease is rare both before puberty as well as after the age of 40 [13]. The mean age of disease presentation was 24.18±5.6 years (14-40). However, the majority of patients 43 (71.7%) presented between the ages of 20-30 years which is consistent with published literature reporting peak incidence in the third decade of life [14]. Additionally, the male to female ratio was 10:1 being consistent with current literature [14, 15].

Increased incidence of pilonidal sinus disease was seen during the Second World War amongst US military personnel and was coined as a ‘Jeep Bottom’ deformity. Similarly, the majority of patients in this study had sedentary occupations and lifestyles. Forty-seven (78.4%) of patients were either drivers, shopkeepers or students thereby reflecting that prolong sitting may increase the risk of pilonidal sinus disease [16].

All the patients were examined before hospital discharge to ensure there were no immediate complications related to the surgical procedure. In Group A, 21 (70%) patients compared to 11 (36.7%) in Group B were discharged within 24 hours of procedure (p-value = 0.001). Therefore, patients who underwent excision and primary repair were discharged significantly sooner than those who underwent Bascom’s operation. No patient in either group required admission greater than 48 hours.

All the patients were followed-up at one week for an assessment of pain and wound review. Patients who underwent Bascom’s repair had significantly less post-operative pain with 23 (76.7%) patients reporting no pain compared to 15 (50%) patients who underwent excision and primary repair (p-value = 0.049). Additionally, those who underwent Bascom’s repair had clean wounds, 28 (93.3%) compared to 23 (76.7%) patients who underwent excision and primary closure (p-value = 0.070). At three-weeks, there were similar results with only one (3.3%) patient who underwent Bascom’s repair showing signs of wound infection compared to 5 (16.7%) patients who underwent excision and primary repair with signs of infection (p-value = 0.085). Although not statistically significant, the results regarding infection rates are similar to existing literature reporting reduced infection rates in Bascom’s repair with only 3 (4%) patients affected [2].

All patients were reviewed 12-weeks post-operatively for signs of disease recurrence. Only one (3.3%) patient who underwent Bascom’s repair compared to five (16.7%) patients who underwent excision and primary closure presented with recurrence of pilonidal disease (p-value = 0.085). Although the results were not significant, they are comparable to previous international studies where the recurrence rate after Bascom’ s technique was 7.3% with 4.6% patients requiring further surgery [2, 17].

Our study is consistent with findings of a recent meta-analysis advocating the preferred use of Bascom’s technique in the treatment of pilonidal sinus disease. In particular, evidence supports Bascom’s is associated with better wound healing and reduced morbidity and therefore should replace the conventional treatment of pilonidal sinus disease [18]. However, the limitations of the study include the small cohort size that made it difficult to adequately power the study to detect a difference. These are typical problems associated with small experimental studies and necessitate the need for further multicenter collaboration. This study was a case series and is reported in line with the PROCESS criteria [19].

## Conclusions

In summary, patients who underwent the Bascom's operation had significantly less postoperative pain and better wound healing. Infection rates and disease recurrence was also less frequent in those whom underwent Bascom’s repair. Therefore, in our patient cohort, we conclude Bascom’s repair appears to be superior to primary excision and repair in reducing patient morbidity.
